# Baicalein reduces the occurrence of cirrhotic endotoxemia by reducing intestinal mucosal apoptosis

**DOI:** 10.1186/s12906-015-0682-8

**Published:** 2015-05-29

**Authors:** Yi Liu, Feng Ye, Wen-jing Zou, Ye Sun, Rui Wang, Ping-ping Han, Zhe Zhang, Xue-liang Yang, Xiaojin Liu

**Affiliations:** Department of Traditional Chinese Medicine, the First Affiliated Hospital of Xi’an Jiaotong University, Xi’an, China; Department of Infectious Diseases, the First Affiliated Hospital of Xi’an Jiaotong University, Xi’an, 710061 China; Department of Geriatrics, the Fifth Hospital of Xi’an, Xi’an, China

**Keywords:** Baicalein, Liver cirrhosis, Endotoxemia, Intestinal mucosa, Apoptosis

## Abstract

**Background:**

The dried roots of Scutellaria baicalensis Georgi, is known in traditional Chinese medicine as Huang Qin (H. qin), and it has been officially and traditionally used in treatment of various diseases such as hepatitis in China. Baicalein (BA), a flavonoid originally isolated from H. qin, has shown a wide range of biological activities. This study was to evaluate whether baicalein, can reduce the intestinal mucosal cell apoptosis caused by cirrhotic endotoxemia and its possible mechanisms.

**Methods:**

For this purpose, compound factors modeling was used to establish endotoxemic cirrhotic rat model. Firstly, we evaluated endotoxin, ALT, AST and TBIL levels after the baicalein treatment (20 mg/kg, i.v.). To investigate the mechanism of baicalein effect on apoptosis, TUNEL assay was used to detect intestinal mucosal apoptosis. RT-PCR was used to detect the expression levels of gene Bcl-2 mRNA and Bax mRNA in intestinal mucosal tissues. Caspase-3 activity of intestinal tissue was detected with colorimetric method in our experiments.

**Results:**

After treatment with BA, the serum endotoxin concentration, the intestinal mucosal apoptosis rate and the activity of caspase-3 of the baicalein group were significantly lower than that of the model and the glutamine group. The serum ALT, AST and TBIL concentration of the BA group were significantly lower than that of the model group. The body weight of the baicalein group was significantly lower than that of the normal group, but it was higher than that of the model group. Among the treatment groups, the mRNA level of anti-apoptotic gene Bcl-2 was the lowest in the model group and the highest in the baicalein group while the mRNA level of pro-apoptotic gene Bax was the lowest in the baicalein group and the highest in the model group.

**Conclusion:**

The present results demonstrated that baicalein could reduce the occurrence of cirrhotic endotoxemia partly by reducing intestinal mucosal apoptosis.

## Background

Cirrhotic endotoxemia is an important factor causing disease progression and multiple organ failures. There are many studies on the formation mechanism of cirrhotic endotoxemia. When cirrhosis was formed, portal hypertension caused hyperemia, edema, and erosion at intestinal mucosa. Intestinal mucosal epithelial cell apoptosis occurred. As a result, intestinal protection functions decreased, and intestinal flora were displaced [1,2]. Then endotoxin went into the blood and endotoxemia was formed, which was considered to be one of the main causes for cirrhotic endotoxemia. Breakdown of intestinal mucosal barrier function in cirrhotic endotoxemia is of great clinical importance [3]. Liver cirrhosis is the initial factor in this mechanism, but one of the key links to endotoxemia formation is the disappearance of intestinal mucosal integrity [4-6]. The present study demonstrated liver cirrhosis was associated with the decrease of intestinal mucosal proliferation and the ratio of proliferation/apoptosis even at the early stage of cirrhosis [7]. Therefore, one of the effective methods in treating cirrhotic endotoxemia might be reducing intestinal mucosal cell apoptosis.

The dried roots of Scutellaria baicalensis Georgi, is known in traditional Chinese medicine as Huang Qin (H. qin), and it has been officially and traditionally used in treatment of various diseases such as hepatitis, pneumonia, jaundice, fever, and acute dysentery in China [8,9]. Baicalein (BA), a flavonoid originally isolated from H. qin, has shown a wide range of biological activities, including antiendotoxic [10], anti-bacterial [11], anti-hepatic fibrosis [11,12], anti-inflammatory [13-17], antiviral [18], etc. Previous studies have demonstrated that BA had cytoprotective and anti-apoptotic effects in myocardial cells [19], liver cells [20,21], lung fibroblasts [22] >and cancer cell lines [23,24]. And it may regulate apoptosis-related genes such as Bcl-2 and BAX etc. [25,26]. And on the basis of this, our study will further explore the anti-apoptotic effect of Baicalein on intestinal mucosal epithelial cells and thus explore whether the occurrence of cirrhotic endotoxemia could be reduced by decreasing intestinal mucosal cell apoptosis.

## Methods

### Experimental animals

90 healthy and clean Sprague Dawley rats were 8 weeks old weighing 190–220 g, and all were purchased from the Laboratory Animal Center of Xi’an Jiaotong University. All animals were housed under controlled temperature (23 ± 1) °C, humidity (55 ± 5) % and 12 h light/12 h dark cycle for 1 week before the experiment.

### Experimental materials

BA (purity 99 %) was purchased from Sigma-Aldrich Chimie Company (Fallavier, France). Glutamine (Kotobuki Pharmaceutical Co., Ltd.) was purchased from the pharmacy of the First Affiliated Hospital of Xi’an Jiaotong University. Carbon tetrachloride analytical reagent (AR) was purchased from Chemical Reagent Branch of Tianjin Zonghengxing Industrial and Trading Co., Ltd. Olive oil (AR) was purchased from Sinopharm Chemical Reagent Co., Ltd. Rat endotoxin quantitative detection kit was purchased from Beijing Jinshanchuan Technology Development Co., Ltd. Apoptosis detection kit was purchased from Biosynthesis Biotechnology Co., Ltd. Total RNA extraction kit was purchased from Takara Biotechnologh (Da Lian) Co., Ltd. Caspase-3 activity detection kit was purchased from Clontech company.

### Reagent preparation

CCL4 oil solution preparation: 40 ml CCL4 was dissolved in 60 ml olive oil and 40 % CCL4 oil solution was prepared and stored at room temperature.

30 % alcoholic beverage preparation: anhydrous ethanol and animal drinking water were prepared in proportion of 3: 7 and stored at room temperature.

Glutamine solution: 15 g glutamine was dissolved 15 ml distilled water and was prepared as 1 g/ml solution.

### The establishment of endotoxemic cirrhotic rat model

Half of male and female Sprague-Dawley rats between 190 and 220 g were obtained from the Animal Research Center of Xi’an Jiaotong University which were maintained according to the Guide for the Care and Use of Laboratory Animals (Institute of Laboratory Animal Resources, 1996, Nat. Acad. Press) and approved by the Xi’an Jiaotong University (Xi’an, China) Institutional Animal Care and Use Committee (IACUC). At the end of experiments, rats were euthanized under Phenobarbital anesthesia.

**Fig. 1:**
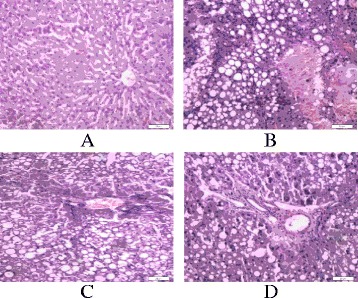
The pathological changes of liver tissue in the each group after treatment (HE staining, 10 × 40). Note: **a** The pathological changes of liver tissue in normal group; **b** The pathological changes of liver tissue in Model group; **c** The pathological changes of liver tissue in Glutamine treatment group; **d** The pathological changes of liver tissue in BA treatment group

Endotoxemic cirrhotic rat model was established through complex factor modeling method using carbon tetrachloride combined with high-fat diet [27,28]. Conventional adaptive feeding of animals was done for 1 week and then an eight-week fibrosis induction period was initiated. All rats were randomly divided into model control group with 80 rats and normal control group with 10 rats. During the initial two-week treatment, 40 % CCL4 olive oil solution was biweekly subcutaneously injected for 0.5 ml/100 g body weight, combined with high-fat diet (20 % lard oil +0.5 % cholesterol +79.5 % corn flour) and 5 % ethanol (White wine and distilled water) as drinking water, followed by two weeks 50 % CCL4 olive oil solution of biweekly subcutaneous injection of 0.3 ml/100 g body weight, with high-fat diet (0.5 % cholesterol +99.5 % corn flour) and 10 % ethanol as drinking water. From the 5^th^ to 8^th^ week, 60 % CCL4 olive oil solution was biweekly subcutaneously injected for 0.3 ml/100 g body weight, combined with high-fat diet (0.5 % cholesterol +99.5 % corn flour) and 10 % ethanol as drinking water (see in Fig. 1). At the 9^th^ week, 5 rats of the model control group were randomly selected, and their liver histopathology and serum endotoxin levels were detected. After the establishment of endotoxemic cirrhotic rat model was determined, follow-up tests were conducted. During modeling, 15 endotoxemic cirrhotic rats died due to liver failure, and the mortality rate was 18 %. No rats died in the normal control group.

### Method of administration

After modeling, 60 rats of the model group were randomly divided into 3 groups, namely BA treatment group, the glutamine treatment control group and the model control group with 20 rats in each group. In all of BA treatment groups, rats were administered for intravenous injection with BA (20 mg/kg [10]); Baicalin were mixed with Poloxamer according to the ratio of 1:5, and then after the mortar was used for grinding for 20 min, 0.9 % NaCl was added. The solution was adjusted to pH 7.4),and the rats were gavaged with 1 ml/100 g aqueous solution for two weeks. 1 ml/100 g glutamine aqueous solution was drenched to the rats of the glutamine group, and 1 ml/100 g aqueous solution was drenched to the rats of model control group. Moreover, the rats in the glutamine and model control group also received an intravenous injection with equivalent volume of 0.9 % saline for 2 weeks.

### Specimen collection and preparation

At 14^th^ day after treatment, i.e. fasting for 24 h after the last administration, specimens were collected, and the specific steps were as the following:

After rat body weight was recorded, 10 % 1 ml/100 g chloral hydrate solution was used for anesthesia by intraperitoneal injection, and then the abdominal cavity was opened under sterile conditions. Under pyrogen-free conditions, abdominal aorta blood was collected into depyrogenation tube and kept for enzyme linked immunosorbent assay (ELISA) detection.

After blood collection, the liver was exposed, and terminal ileum tissue of about 3 cm was taken from the intestinal tract. One part of this intestinal tissue was put in 4 % paraformaldehyde solution for TUNEL assay,and the other part was put in liquid nitrogen for later use.

### Detection of serum endotoxin levels with two-step double antibody sandwich enzyme-linked immunosorbent assay

The enzyme linked immunosorbent assay (ELISA) was used for the detection and quantification of serum endotoxin levels of rats in all groups according to the manufacturer’s description (range 2.5–80 pg/mL). Samples of serum (100 μL) were dispensed into wells of 96-well microlitre plates which had been pre-coated with rat endotoxin (LPS) antibody. After a 30 min incubation at 37 °C, unbound proteins were washed away from the wells. A protein-specific biotinylated antibody was then added and the plates were incubated for 2 more hours at 37 °C. After further rinsing to remove unbound antibody, a substrate solution A, B was added to each well and the mixture was incubated for 20 min at 37 °C. The reaction was terminated with the addition of a stop solution. A value was read at 450 nm in microplate reader. With a value as vertical coordinates and the standard concentration as abscissa, standard curve was drawn. According to the value of serum sample, its concentration was found at the standard curve, and the data were expressed as mean value.

Serum ALT, AST and TBIL levels were detected with ELISA method.

### Detection of intestinal mucosal cell apoptosis by TUNEL

Terminal deoxynucleotide transferase dUTP nick end labeling (TUNEL) was performed to detect cellular apoptosis on terminal ileum tissue using in situ cell death detection Kit according to the manufacturer’s instructions. Sections were post-fixed in ethanol-acetic acid (2:1) and rinsed. Then the sections were incubated with proteinase K (100 μg/mL), rinsed, incubated in 3 % H_2_O_2_, and washed with PBS for 10 min followed by washing with permeabilization solution (0.1 % Triton X-100, 0.1 % sodium citrate) for 5 min. After the sections were washed twice with PBS, incubated in TUNEL reaction mixture,and rinsed again. Sections were visualized using converter-POD with 0.02 % 3,3’-diaminobenzidine (DAB). Mayer’s hematoxylin was used for counter-staining. The sections were finally mounted onto gelatin-coated slides and then they were air dried overnight at room temperature. Yellow stained nucleus was TUNEL positive cells.

TUNEL-positive cell quantitative method was used. In each group, 5 cases of well-stained sections were selected and magnified 10 times for observation under the microscope objective. 5 high power fields (×400) in each section were randomly selected, and a total of 25 high power fields were photographed into computer (resolution is 800× 600). Q550CW image signal acquisition and analysis system was employed to calculate intestinal epithelial cell apoptosis index. The number of apoptotic cells / the total cell number × 100 % = the apoptosis rate of this field, and the average rate of apoptosis of five fields was the apoptosis rate of rat intestinal mucosal cells in the group.

### Detection of apoptotic gene Bcl-2 mRNA and Bax mRNA by RT-PCR

#### Experimental procedures

Primer synthesis: According to Complementary DNA (cDNA) sequence of Bcl-2, Bax and β-actin, two pairs of primers were designed respectively. Bcl-2 upstream primer was 5’-GAACTGGGGGAGGATTGTGG-3 ’, and its downstream primer was 5’-CCACCGAA CTCAAAGAAGG-3’, and the product length was 269 bp; Bax upstream primer was 5’-GTTACAGGG TTTCATCCAGG-3 ’, and its downstream primer was 5’-CAAAGTAGAAGAGGGCAACC-3, and the product length was 272 bp; The upstream primer of housekeeping gene β-actin was 5’-AACCCT AAG GCC AAC CGT GAA AAG-3 ’, and its downstream primer was 5’-TCATGAGGTAGTCTGTCAG-3’, and the product length was 241 bp. All primers were designed and synthesized by Beijing Aoke Company.

Total RNA extraction: After the rats were sacrificed, dry roasted tweezers were used to clip terminal ileum tissue of about 3 cm quickly from the intestinal tract. Intestinal contents were rinsed with saline, and the tissues were cut into 2 cm × 1 cm size and they were cooled and preserved rapidly with liquid nitrogen. In the experiment, 100 mg colon specimens were taken, and the tissues were ground into power in liquid nitrogen. Total RNA was extracted by Trizol reagent extraction method with reference to the method on the kit.

RNA was reverse transcribed into cDNA. Reverse transcription reaction was performed according to Invitrogen’s Superscript III First-Strand Synthesis reverse transcription kit instructions. The product obtained was stored at −20 °C or used directly.

Amplification of the housekeeping genes: PCR reaction system (0.5 μl Taq DNA polymerase (5u/μl), 5 μl 5 × Buffer (1.5 mmol/L MgCl_2_), 1 μl P1 (100 μmol/l), 1 μl P2 (100 μmol/l), 0.5 μl dNTP (10 mmol/l), 10 μl template cDNA, 4 μl 50 mmol/L MgCl_2_, 28 μl H_2_O, and the total reaction system was 50 μl). PCR reaction amplification conditions: initial denaturation: 95 °C 30 s, denaturation: 95 °C 30 s, renaturation: 58 °C 30 s, extension: 72 °C 30 s, 35 cycles, 72 °C for 10 min.

PCR amplification of gene fragments: reaction system (0.5 μl Taq DNA polymerase (5u/μl), 5 μl 5 × Buffer (1.5 mmol/L MgCl_2_), 1 μl P1 (100 μmol/l), 1 μl P2 (100 μmol/l), 0.5 μl dNTP (10 mmol/l), 10 μl template cDNA, 4 μl 50 mmol/L MgCl_2_, 28 μl ddH_2_O, and the total reaction system was 50 μl). PCR reaction amplification conditions: initial denaturation: 95 °C 5 min, denaturation: 94 °C 45 s, renaturation: 56 °C 45 s, extension: 72 °C 45 s, 35 cycles, 72 °C for 10 min.

Electrophoresis was performed to PCR products in a 2 % agarose gel at 80 V for 30 min. Then PCR products were observed and photographed in the gel imager, and the Quantity One analysis system was used for the analysis of electrophoretic bands to calculate the values of Bcl-2 products, Bax products and β-actin products, that is, Bcl-2 and Bax mRNA expressions in tissues.

The above process was repeated three times.

### The detection of caspase-3 activity of small intestinal tissues with colorimetric method

100 mg fresh terminal ileum tissue was homogenized and centrifuged, and protein concentration was determined and adjusted. DTT (1 mol/L 10 μl) of Caspase-3 detection kit and 50 μl 2 × reaction buffer were added into 10ul supernatant sample protein, and then 10 μl 1 mmol Caspase-3 substrate Ac-DEVD-pNA was added. The mixture was incubated at 37 °C for 60 min. The fluorescence units of Caspase-3 before and after incubation were determined with excitation wavelength 400 nm and emission wavelength 405 nm according to the instruction of the kit. Lysis Buffer and reaction Buffer were taken as the blank control.

### Statistical methods

SPSS13.0 statistical analysis software package was used. One-way analysis of variance (one-way ANOVA) was performed, and q test (Student-Newman-Keuls test, S-N-K) was used for pair wise comparisons. Data were all expressed with ($\overline{x}\pm s$), and when heterogeneity of variance appeared, the data were square-root transformed. P <0.05 was considered to be statistically significant.

## Results

### Verification of endotoxemic cirrhotic rat model

During modeling, 15 rats of endotoxemic cirrhotic rat model died due to liver failure, and the mortality rate was 18 %. No rats died in the normal control group.

After modeling, five rats were randomly selected from the model rats, and all had liver pathological examinations and serum endotoxin level detection. Each of them showed different degree of liver cirrhosis. Pathological results showed that hepatic lobule structure was damaged in model rats. Liver cell cords were no longer radiating formation but arranged disorderly with obvious swelling and degeneration --mostly fatty degeneration. And the necrosis of some liver cells occurred, and inflammatory cell infiltration was obvious (see in Fig. 1b). And for five model rats, the mean detection level of endotoxin was 67.4 ± 22.6 pg/ml, and it was higher than that in the formation of endotoxemic cirrhotic model reported in the literature [29], and it was also higher than that of the normal control group. This confirmed the modeling success of endotoxemic cirrhotic model.

After treatment, we also examined the liver pathological changes of the BA and the glutamine treatment groups. In the BA and the glutamine treatment groups, part of the structure of hepatic lobule was not complete. And the necrosis of some liver cells and inflammatory cell infiltration seemed to be relatively minor in the BA than that in the glutamine treatment group (Fig. 1c and d).

**Fig. 2:**
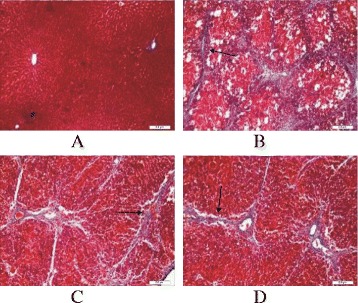
The pathological changes of liver tissue in the each group after treatment (Masson’s trichrome stain, ×10). Note: **a** The pathological changes of liver tissue in normal group; **b** The pathological changes of liver tissue in Model group; **c** The pathological changes of liver tissue in Glutamine treatment group; **d** The pathological changes of liver tissue in BA treatment group

In Masson staining, blue color was hyperplastic collagen fibers. In the normal group, rats’ hepatic lobule structure was clear. In the model group, large amount of blue hyperplastic thick collagen fibers were observed in portal area and lobules. Hepatic cell cords of liver lobule were arranged in disorder and a large amount of unequal-sized pseudolobules were formed. In the BA group and the Glutamine group, lesion degree was similar, collagen fiber was less than that of the model group, the central vein stellate was thickening, and some fibrous septum in portal area was formed, but fibrous septum was thinner than that of the model group. (Fig. 2)

### Comparison of body weight of rats after intervention

Compared with the normal control group, body mass of rats in the model group decreased significantly (P < 0.05). Compared with the model group, body mass of rats in the baicalin group significantly increased (P < 0.05). In the glutamine group, body mass of rats also increased, but the difference was not statistically significant (Table 1).

**Table 1:** Comparison of body weight of rats after intervention (g) ($\overline{x}\pm s$_,__g_)

Groups	Number	body weight
Normal control group (NG)	10	426.11 ± 32.43 ^b^
Model group (MG)	20	210.31 ± 18.45 ^a^
Glutamine group (GG)	20	249.52 ± 16.70 ^a^
BA group (BAG)	20	295.61 ± 23.48 ^a b^

*F*_endotoxin_ = 52.89, *F*_ALT_ = 223.49, *F*_AST_ = 223.49, *F*_TBIL_ =234.90

^a^Compared with the normal control group, *P* < 0.05

^b^Compared with the model group, *P* < 0.05

^c^Compared with the glutamine group, *P* < 0.05

### Comparison of endotoxin, ALT, AST and TBIL levels in each group after intervention

Compared with the normal control group, the endotoxin levels of all of experimental groups significantly increased, (*P* <0.05). The serum endotoxin levels of the model group increased from 67.4 ± 22.6 pg/ml at the end of modeling to 115.12 ± 45.68 pg/ml, and it was significantly higher than that of the BA treatment group, (*P* <0.05). It was also higher than that of the glutamine group, but there was no significant difference. The serum endotoxin level of the BA treatment group was significantly lower than that of the glutamine treatment group, (*P* <0.05) (Table 2).

**Table 2:** Comparison of blood endotoxin, ALT, AST and TBIL levels of rats in each group ($\overline{x}\pm s$, Pg/ml, U/L, U/L, μmol/L)

Group	N	Endotoxin	ALT	AST	TBIL
NG	10	3.78 ± 2.47 ^b^ ^c^	28.21 ± 3.02 ^b c^	26.83 ± 6.12 ^b^	6.93 ± 0.29 ^b c^
MG	20	115.12 ± 46.86 ^a^	117.63 ± 15.82 ^a c^	192.61 ± 29.65 ^a^	24.51 ± 3.29 ^a^
GG	20	96.29 ± 39.20^a^	75.12 ± 5.02 ^a b^	83.84 ± 5.88 ^a b^	13.82 ± 0.73 ^a b^
BAG	20	38.27 ± 4.67^bc^	71.21 ± 4.02 ^a b^	86.43 ± 6.28 ^a b^	13.75 ± 0.86 ^a b^

*F*_endotoxin_ = 52.89, *F*_ALT_ = 223.49, *F*_AST_ = 223.49, *F*_TBIL_ =234.90

^a^Compared with the normal control group, *P* < 0.05

^b^Compared with the model group, *P* < 0.05

^c^Compared with the glutamine group, *P* < 0.05

Compared with the normal control group, the ALT, AST and TBIL levels of all of experimental groups significantly increased, (*P* <0.05). The serum ALT, AST and TBIL concentration of the BA treatment group were significantly lower than that of the model group, (*P* <0.05) (Table 2).

### Detection of intestinal mucosal cell apoptosis in each group by TUNEL

Intestinal mucosal cell apoptosis in normal rats was seen occasionally (Fig. 2a). The intestinal mucosal cell apoptosis of endotoxemic cirrhotic rats significantly increased. The positive nuclei were brown or black, and were scattered diffusely in the field. The nuclei were pyknotic-like, or chromatin aggregated to the surrounding, or the nuclei were fragmentary (Fig. 2b). The intestinal mucosal cell apoptosis of the BA group and the glutamine group increased compared with that of the normal control group, and the apoptotic nuclei were brown or black, but the intestinal mucosal cell apoptosis in these two groups significantly improved compared with that in the model group, and the improvement in the BA group was the most significant (Fig. 2c and d).

The intestinal mucosal cell apoptosis rate of the experimental groups was significantly higher than that of the normal control group, (*P* < 0.05). The intestinal mucosal cell apoptosis rate of the model group was significantly higher than that of the BA treatment group and the glutamine group, (*P* < 0.05). The intestinal mucosal cell apoptosis rate of the BA group was significantly lower than that of the glutamine group, (*P* <0.05) (Table 3).

**Table 3:** The intestinal mucosal cell apoptosis rate of rats in each group ($\overline{x}\pm s$, %)

Groups	Rat number	Apoptosis rate
Normal control group (NG)	10	4.80 ± 1.92^bc^
Model group (MG)	20	48.80 ± 6.98^ac^
Glutamine group (GG)	20	16.00 ± 2.92^ab^
BA group (BAG)	20	12.80 ± 2.17^abc^

*F* = 339.39

^a^Compared with the normal control group, *P* < 0.05

^b^Compared with the model group, *P* < 0.05

^c^Compared with the glutamine group, *P* < 0.05

### Comparison of Bcl-2 mRNA level, Bax mRNA level and the activity of caspase-3 in each group

**Fig. 3:**
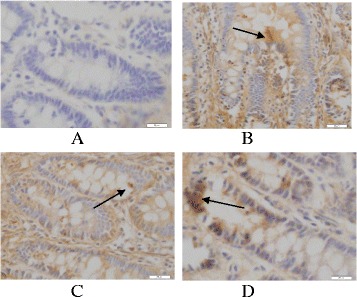
The intestinal mucosal cell apoptosis in the each group after treatment (TUNLE staining, 10 × 40). Note: **a** The pathological changes of liver tissue in normal group; **b** The pathological changes of liver tissue in Model group; **c** The pathological changes of liver tissue in Glutamine treatment group; **d** The pathological changes of liver tissue in BA treatment group

RT-PCR reactions showed there was internal reference β-actin band at 241 bp in each group (Fig. 3a). RT-PCR results showed that there was a specific Bcl-2 band at 269 bp (Fig. 3b), and quantitative values were shown in Table 4. Bcl-2 mRNA levels of intestinal mucosal tissues in the experimental groups significantly decreased compared with that of the normal control group, (*P* <0.05). Bcl-2 mRNA levels of intestinal mucosal tissues in the model group were significantly lower than that of the BA treatment group and the glutamine treatment group, (*P* < 0.05). Bcl-2 RNA level of intestinal mucosal tissue in the BA treatment group was significantly higher than that of the glutamine group, (*P* <0.05).

**Table 4:** Bcl-2 mRNA relative expression levels of intestinal mucosal tissues in each group ($\overline{x}\pm s$_,_ copies/ml)

Groups	N	*Bcl-2 mRNA*	Bax mRNA
Normal control group (NG)	10	1.73 ± 0.12^bc^	0.52 ± 0.12^bc^
Model group (MG)	20	0.50 ± 0.13^ac^	1.87 ± 0.11 ^ac^
Glutamine group (GG)	20	0.76 ± 0.13 ^ab^	0.79 ± 0.14^ab^
BA group (BAG)	20	0.98 ± 0.11^abc^	0.66 ± 0.13^abc^

*F*_*Bcl-2*_ = 10.48, *F*_Bax_ = 20.41

^a^Compared with the normal control group, *P* < 0.05

^b^Compared with the model group, *P* < 0.05

^c^Compared with the glutamine group, *P* < 0.05

RT-PCR results showed that there was a specific Bax band at 272 bp (Fig. 3c), and the quantitative values were shown in Table 4. Bax mRNA levels of intestinal mucosal tissue in the experimental groups significantly increased compared with that of the normal control group, (*P* <0.05). Bax mRNA level of intestinal mucosal tissue in the model group was significantly higher than that of the BA treatment group and the glutamine treatment group, (*P* <0.05). Bax mRNA level of intestinal mucosal tissue in the BA treatment group was significantly lower than that of the glutamine group, (P < 0.05).

**Fig. 4:**
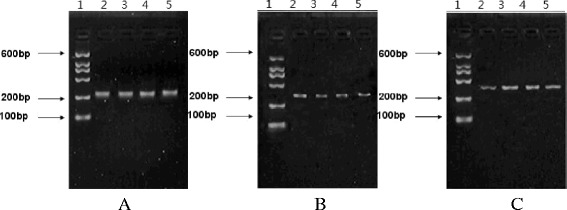
mRNA expression of β-actin, Bcl-2 and Bax in each group. Note: Lanes: 1 marker; 2 normal control group; 3 model group; 4 glutamine group; 5 BA treatment group. **a** β-actin mRNA expression in each group; length of target product: 241 bp. **b** Bcl-2 mRNA expression in each group; length of target product: 269 bp. **c** Bax mRNA expression in each group; length of target product: 272 bp

After treatment with BA, the activity of caspase-3 in the intestinal mucosal of the baicalein group was significantly lower than that of the model and the glutamine group, (P <0.05) (Table 5) Fig. 4.

**Table 5:** Changes of activity of caspase-3 in intestinal mucosal tissues in each group ($\overline{x}\pm s$)

Groups	N	caspase-3
Normal control group (NG)	10	289.11 ± 34.75^bc^
Model group (MG)	20	1076.23 ± 28.71^ac^
Glutamine group (GG)	20	578.35 ± 96.37^ab^
BA group (BAG)	20	420.21 ± 44.81^abc^

*F*_caspase-3_ = 525.39

^a^Compared with the normal control group, *P* < 0.05

^b^Compared with the model group, *P* < 0.05

^c^Compared with the glutamine group, *P* < 0.05

## Discussions and conclusion

Intestinal mucosal cells are the body’s first line of defense against endogenous substances such as bacteria, viruses and endotoxin. Under normal circumstances, intestinal mucosal cells update once every 3–5 days, and this balance process is maintained through apoptosis and regeneration of intestinal mucosal cells. Once the apoptosis of intestinal mucosal cells is greater than their proliferation and then the intestinal barrier function is damaged, so a large number of exogenous substances including endotoxin enter the body to form endotoxemia [30]. Liver cirrhosis is associated with decreased intestinal mucosal proliferation and enterocyte apoptosis even at early stages of cirrhosis liver disease [7]. Accumulating experimental and clinical evidences suggest that intestinal barrier dysfunction and subsequent gut-derived endotoxemia represent an important common pathogenetic mechanism in the development of diverse complications of cirrhosis [31]. It is also possible that a cycle develops between intestinal damage and liver injury through the portal circulation [32,33]. Thus the vicious cycle of endotoxemia - intestinal mucosal injury is formed to promote disease progression. When the condition of liver cirrhosis can not be changed, the integrity of intestinal mucosa has become the key to the treatment of disease [16,17].

Scutellaria root is dried root of Labiatae perennial herb – Scutellaria baicalensis Georgi, and its nature is bitter cold. It can enter the lung, stomach, gall bladder and large intestine channels. It has the function of clearing heat, eliminating damp, purging fire and removing toxin in traditional Chinese medicine. In clinical applications, it has been found that BA preparation has certain clinical efficacy on endotoxemic cirrhotic patients, and basic researches also confirmed that BA extracts could protect liver cells and had anti-endotoxin function [16,17].

Our experiment showed that intestinal mucosal apoptosis rate of cirrhotic rats was significantly higher than that of the normal control group. And after treatment with BA, we found that the intestinal mucosal apoptosis rate of cirrhotic rats and the endotoxin level were decreased. Though with the current study design, it is difficult to determine whether intestinal mucosal cell apoptosis is closely related with the formation of endotoxemia. And unfortunately, so far, no relevant studies could directly prove a direct causal relationship between endotoxin and intestinal mucosal cell apoptosis. But no matter which happens first, BA undoubtedly contributes to the treatment of cirrhotic endotoxemia to reduce intestinal mucosal apoptosis and reduce endotoxin levels.

Bcl-2 family is a group of apoptosis-related genes, including Bcl-2, Bal-xl, Bcl-xs, Bax, etc. Bcl-2 and Bax is a pair of genes that are most closely related with apoptosis, and both genes are present as homodimers respectively, and they can also form heterodimers [34,35]. When intracellular Bax is highly expressed, cells are sensitive to death signals and cell apoptosis is accelerated. When Bcl-2 is highly expressed, Bcl-2 can form heterodimers with Bax to inhibit apoptosis [36,37]. When Bax homodimers are formed, they will induce apoptosis. With Bcl-2 expression increasing, more and more Bax dimers separate to form more stable Bax-Bcl-2 heterodimers with Bcl-2 than Bax-Bax, thereby inhibiting Bax-Bax induced apoptosis, and therefore the ratio of intracellular Bax to Bcl-2 is an important factor in the regulation of apoptosis.

In our study RT-PCR results showed that the mRNA level of anti-apoptotic gene Bcl-2 was the lowest in intestinal mucosal tissues of the model group while it was the highest in the BA treatment group among the treatment groups; while the mRNA level of pro-apoptotic gene Bax was the lowest in the BA group while it was the highest in the model group among the treatment groups (Fig. 4). There are similar results in the treatment of other diseases by BA [14,26]. This result indicated that BA could reduce the expression of pro-apoptotic gene Bax, and simultaneously increase the expression of anti-apoptotic gene Bcl-2, and finally might improve the ratio of Bcl-2/Bax to inhibit the apoptosis of intestinal mucosal cells, thereby reducing the formation of endotoxemia.

Some evidences have showed that the glutamine with protective effect on intestinal mucosa barrier injury can reduce the apoptosis of intestinal mucosal epithelial cells [38-41]. So in our study it was made as a control group. The Caspases family protease is core components of apoptotic pathways. Caspase-8, Caspase-9 and Caspase-12 respectively act as molecular switch in the pathway of cell surface death receptor (extrinsic pathway), the mitochondrial /Cyt-c pathway (intrinsic pathway) and the endoplasmic reticulum pathway. Caspase-3, as the apoptosis effector proteins, is a common downstream pathway for three ways of cell apoptosis, and it plays a decisive role in the apoptosis signal transduction pathway. So we studied the changes of intestinal mucosal Caspase-3 after the treatment by baicalin, and the results showed that Caspase-3 was decreased after the treatment in both the baicalin treatment group and the glutamine group. Current studies suggest that for apoptosis, there exists three pathways such as extrinsic pathway, intrinsic pathway or mitochondrial pathway and endoplasmic reticulum pathway [42-44], and these three kinds of pathways are interconnected, so it is very complex. So what is really the pathway through which baicalin alleviated intestinal epithelial cell apoptosis, which is still in our research.

In our study, the apoptosis rate of intestinal mucosal epithelial cells and the serum endotoxin levels in the BA treatment group were significantly lower than that of the glutamine treatment group, which suggested that BA had a stronger ability to protect intestinal mucosal cells and to make endotoxin levels decrease. More interesting things were that in Table 2 the intestinal mucosal apoptosis in both the glutamine group and the BA group was significantly reduced compared to the model control group, yet in Table 1 it appeared that endotoxin levels were quite different between the glutamine and BA treatment groups. While there appears to be a significant difference in intestinal apoptosis between the two groups, it seems very minor, and much too small in relation to the difference in endotoxin levels. Previous reports have demonstrated that BA has shown a wide range of biological activities, including antiendotoxic [10], anti-bacterial [11] and anti-hepatic fibrosis [12,13]. That might be the reason. In other words, Baicalin might reduce endotoxin levels through a variety of ways, not just to reduce the intestinal mucosal damage by reducing the apoptosis. Thus it makes the baicalin much more hopeful in treating cirrhotic endotoxemia.

Our study investigated the treatment effect of BA on cirrhotic endotoxemia only from the perspective of apoptosis, and more complete and in-depth results also needs further research. But it showed that baicalein might be a very promising drug in treating cirrhotic endotoxemia.
